# Developing a values-based interorganisational Care at the End-of-Life Collaborative framework for the Australian context: A mixed-methods, practice-based research protocol

**DOI:** 10.1177/26323524251337491

**Published:** 2025-05-08

**Authors:** Carol Hope, Leah East, John Rosenberg, Melissa Taylor

**Affiliations:** 1 University of Southern Queensland – Ipswich Campus, QLD, Australia; 2 University of the Sunshine Coast, Sippy Downs, QLD, Australia

**Keywords:** Collaborative, care at the end of life, palliative care, interorganisational collaborative, mixed-methods case study, Delphi methodology, values-based collaborative, research protocol

## Abstract

**Background:**

As the population ages and the demand for quality care at the end of life increases, health costs are increasing, thus creating a perfect storm of need, outstripping the supply of palliative care services. Engaging communities to support care at the end of life through the establishment of values-based interorganisational Care at the End-of-Life Collaboratives is one approach to addressing this problem. Whilst there is an abundance of literature about collaboratives, understanding of those supporting care at the end of life is lacking.

**Objectives:**

The objectives of this research are to understand the contextual factors using an evaluation framework that support the success of values-based interorganisational Care at the End-of-Life Collaboratives to achieve system improvements at a regional level and develop a framework for establishing sustainable Care at the End-of-Life Collaboratives in jurisdictions across Australia.

**Design:**

This is a two-phase study that uses a mixed-methods case study and a Delphi methodology.

**Methods and analysis:**

Phase I data collection is based on the RE-AIM framework, which uses the Partnership Self-Assessment Tool (PSAT) and semi-structured interviews with Collaborative members and key stakeholders. A review of collaborative documentation, including meeting minutes and reports, will also be completed. Phase II will include a minimum of two surveys of the expert group recruited from the peak palliative care bodies across Australia. Quantitative data in this study will be analysed using descriptive statistics and frequency distributions. A reflexive approach to content analysis of qualitative data will be adopted.

**Ethics:**

This research is approved by the University of Southern Queensland Human Research Ethics Committee (approval ETH2023-0718).

**Discussion:**

Understanding the contextual factors that contribute to the sustainability of an existing Care at the End-of-Life Collaborative within Australia will enable the foundation of a framework for developing similar collaboratives, for refinement through expert consensus using Delphi methodology.

## Background

Public knowledge and awareness of palliative care in the community remains low. Internationally, substantial health planning challenges exist, and these are exacerbated by rapidly ageing populations and increasing health costs, all of which can impact care at the end of life. In this research, care at the end of life refers to care or support provided to people approaching the end of life due to any reason or cause. In the health literature, care at the end of life is usually labelled as palliative care. A recent literature review demonstrates that the public’s knowledge and awareness of palliative care have remained low despite growth in the sector.^1^ Globally, surveys of the public’s understanding of palliative care indicate between 19% (in Northern Ireland) and 70% (in the United States) of the public have no knowledge of palliative care.^2^ According to McIlfatrick et al., the public is ‘death denying’ and conversations about death and dying are largely unwelcome.^3^ Research continues to show that the public associates palliative care with cancer^3,^4^^ and giving up.^5^ It is widely documented that most people’s understanding and opinions about palliative care depend on their personal experience or what the media reports.^1,6,7^ Relationality exists with popular beliefs that palliative care is widely about pain relief^8^ and medical interventions for the last few days of life.^1,8,9^

Mannix describes that death was a community experience until the second half of the 20th century, where dying people were cared for at home by family and the broader community.^7^ Mannix further contends that the observance of death enabled people to recognise its signs and patterns. The advancement of medical knowledge and technology has stripped society of its understanding of dying, repositioning death from inevitable to a failure.^7^

Whilst death literacy in communities is decreasing, there is evidence that home is the preferred place of death remains high in Australia. In the absence of population-based data describing the preference for a place of death, the Productivity Commission contends the best available estimate is that up to 70% of Australians would prefer to die at home.^10^ However, proximity to death is a factor in determining preferred place of death, with one Australian study concluding preference for a home death falls from 90% to 52% in the last week of life, which may be due to the increase in symptoms and caregiving responsibilities required to remain at home.^11^ The international literature supports this, with a systematic review finding that 31%–87% of people would prefer to die at home, indicating heterogeneity is a factor in determining preferred place of death.^12^ Despite acknowledgement that a high proportion of people would prefer to die at home in Australia, only 4%–12% of people achieve this, with 49% of all deaths in Australia occurring in the hospital-admitted inpatient settings and 36% occurring in residential aged care settings.^13^ The contrast between where palliative care services are delivered and where people want to die is further highlighted in activity figures, which show only 20% of palliative care activity occurs outside the hospital setting, and investment in community palliative care services is less than 2% of the total cost of death in Australia.^13^

In seeking to improve the experience of death and dying across communities, a broader approach to care at the end of life is needed. According to Fliedner, new strategies to engage the public in palliative care initiatives should be established, including community interventions and public health approaches which integrate care at the end of life across health and care systems.^8^ The emergence of the public health approach to palliative care over the last 20–25 years has shifted the dial away from a one-way approach (professionals at the centre of care) towards a community-centric approach.^14^ Such public health palliative care models have demonstrated effectiveness across the globe.^15–17^

The proposed study examines a novel approach to improving care at the end of life at a local system level, through the establishment of a regional Australian interorganisational values-based Care at the End-of-Life Collaborative (‘the Collaborative’). The Collaborative was established in 2018 to address the lack of coordination and collaboration between the ecosystem of service providers and community groups that support and deliver care to people approaching the end of life in the region. The Collaborative brings together stakeholders from across the spectrum of community, health and social care to optimise systems, processes and outcomes for people approaching the end of life in the community. Member organisations and individuals include (but are not limited to) specialist palliative care providers, hospital services (including emergency department and intensive care unit representatives), primary care organisations (including General Practitioners), residential aged care homes, community aged care providers, community groups, consumers and peak body organisations.

The values-based context of the Collaborative is derived from the type, scale and enablers of integration described in the Rainbow Model of Integrated Care.^18^ The Collaborative involves organisational integration, targeting a subgroup of the population (those approaching the end of life). Linked inherently with the original triple aim of integrated care, the Collaborative focuses on better outcomes and improving experiences. This is consistent with normative integration, which Valentijn et al. describe as ‘the development and maintenance of a common frame of reference (i.e. shared mission, vision, values and culture) between organisations, groups and individuals’ (p. 3).^19^ The Collaborative has adopted a Charter as its partnership instrument, rather than more formal instruments such as memorandum of understanding or partnership agreement. The Charter defines the Collaborative’s purpose, focus, objective and guiding principles. Therefore, to distinguish it from structured, governance-based collaborative models, the Collaborative in this study is described as values based and is an intersection of organisations and community that is collectively focused on quality, multidimensional, person-centred care at the end of life for all members of the community.

Despite the global growth in research about effectiveness of interorganisational collaboratives and networks, knowledge and understanding of such entities are largely theoretical rather than grounded in empirical research.^20^ Understanding of how values-based interorganisational collaboratives function and key success factors is lacking, thus further research is needed.^21,22^ This is exacerbated by a dearth of research regarding interorganisational collaborations specifically focused on care at the end of life. In this study, the functions and success of the Collaborative will be evaluated in relation to its Charter, which includes purpose, focus and objective of the Collaborative.

## Research design

This protocol describes a two-phase study; phase I uses a mixed-methods case study design, and phase II employs a Delphi method to achieve expert consensus of a model for a Care at the End-of-Life Collaborative for implementation across Australia.

### Research question and objectives

This study has two research questions, one for each phase of the study.

### Phase I research question

• How does a values-based, interorganisational collaborative approach to care at the end of life in a defined geographical region achieve the aim of improving the systems of care at the end of life for all?

Specific areas to be explored include the success of the Collaborative to achieve its aims (system improvements), the capacity of the Collaborative to deliver its agreed actions, and the impacts of the informal evolution (values-based nature) of the Collaborative. The outcomes and results from phase I of the study will be used to inform phase II.

### Phase II research question

• What are the guiding principles and framework that best support the establishment of values-based interorganisational Care at the End-of-Life Collaboratives in other regions across Australia?

### Theoretical foundations

Aligning the theoretical foundation and paradigm to the research questions is critical to ensuring accurate and relevant findings.^23^ The ontological perspective adopted for this research is realism, which asserts there is an external reality that exists independently of individuals’ beliefs or understanding about it.^24^ Realist ontology posits that there is a single, tangible reality that can be measured and understood through empirical evidence.^25^ Auschra and Aunger et al. contend that studying interorganisational collaborations through a realist lens helps us to understand how and why they work, especially in complex adaptive systems like health.^26,27^ In research about interorganisational collaborations, realists produce causal explanations in the context of unobservable structures, processes and mechanisms which, in the context of this study, works with the premise that the success, mechanisms and outcomes of interorganisational collaborations are highly contextual. Seeking to understand mechanisms within the specific context will enable an explanation of how the Collaborative works. The understanding of how and why interorganisational collaborations work is described in the literature as ‘the black box’, and a realist approach to the methodology is the ideal approach to unpacking this ‘black box’.^28^ A realist perspective will help the researchers in this study to holistically understand the structures and processes of the interorganisational Care at the End-of-Life Collaboration that is the subject of this study.

Pragmatism is the epistemological perspective that underpins the methodology of this study, as it supports a problem-centred approach by which the phenomenon can be explained by both theory and real-world practice simultaneously. According to Snape and Spencer, pragmatism is the ‘toolkit’ approach to research, which enables researchers to choose appropriate methods to address the research question, rather than focusing on a specific philosophical stance.^28^ This agnostic approach to methodology enables the researchers to design data collection based on what works to address the research question. In contrast, a positivist approach would require a specific hypothesis to deductively make and test predictions of results and uses a reductionist stance through objective measurements to determine cause and effect.^25^ Similarly, post-positivism believes in an objective reality, but it also acknowledges that the research process is influenced by value judgements of researchers.^25^ Neither positivist nor post-positivist perspectives are appropriate for this study, as they do not enable the deep and holistic understanding of the Collaborative required for phase II of the study. The final output of this study will be a Care at the End-of-Life Collaborative model specifically developed for practical application in Australia. Pragmatism takes a problem-centred, pluralistic approach to achieve real-world practice solutions.^29^

Alternatively, constructivism and interpretivism seek to understand through social and historical construction to achieve theory generation.^29^ Constructivism builds knowledge through interaction between the researcher and the participants, whilst interpretivism views the researcher as a collaborator who engages participants to develop knowledge and insights.^25^ Adopting a singular constructivist or interpretivist perspective for this study’s methodology would be problematic due to the researcher’s historical role in the Collaborative being studied. As part of the pragmatic approach to the research methodology, some elements of data analysis in this study will take a constructivist perspective. For example, the methods include semi-structured interviews of Collaborative members during phase I. Analysis of these data will enable the researcher to understand the perspectives of the members regarding their experiences in being part of the Collaborative. These data will be analysed and triangulated with other data (quantitative and qualitative) to gain a holistic and deep understanding of the factors that influence the Collaborative’s success and inform the first iteration of a Care at the End-of-Life Collaborative model to commence phase II of the study.

### Research methodology

This study is being undertaken as part of a Doctor of Professional Studies program at the University of Southern Queensland, Australia. Often referred to as ‘practice-based’ or ‘profession-specific’ doctorates, such programs are described by Costley and Lester as ‘more closely geared to practising professionals undertaking research and development in the workplace’ (p. 257).^30^ Consistent with the program requirements, the research design presented in this article is a practice-based study which encourages independent learning through work. The benefits of practice-based study derive from the triple dividend, as described by Fergusson et al., including for the researcher (personal outcomes), organisational (workplace outcomes) and knowledge (academic/professional outcomes).^31^

The methodology of this study aligns with a practice-based approach and will adopt a mixed-method case study design that combines qualitative and quantitative research methods.^29^ The phenomenon being studied in this practice-based case study research (the Collaborative) is a multifaceted system, and thus requires a practical and logistically focused, mixed methodology to achieve a comprehensive understanding of the phenomenon and to answer the questions posed by this research.

### Ethical considerations

This study has received approval from the University of Southern Queensland’s Human Research Ethics Committee as a low-risk study. Ethical consideration has specifically been acknowledged as two of the researchers are known to the participants in a professional capacity, through their membership of the Collaborative or other previous projects. Currently, two of the researchers are co-Chairs of the Collaborative.

To mitigate the potential for coercion to participate and potential bias, the researchers will not be involved in recruitment, communication or data collection for phase I of this study. A third-party media and marketing organisation and an experienced interviewer will be employed to recruit participants, undertake the semi-structured interviews and redact transcripts of identifying information. The researchers will undertake all data analysis. Two of the four researchers undertaking this study have had no prior involvement in the Collaborative and will review all analyses for potential bias.

## Methods

**Figure 1.**
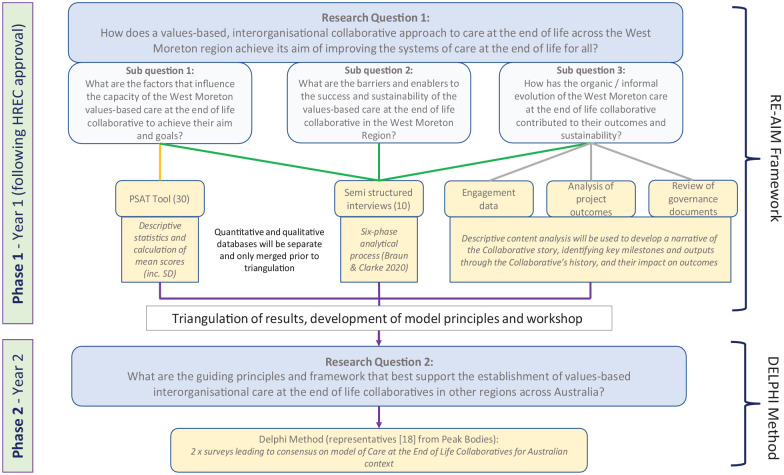
Study methodology.

Figure 1 shows the overall study methodology linked to the research questions, data collection and data analysis.

### Phase I

Consistent with the pragmatist paradigm, phase I of this study employs a philosophically agnostic methodology. A mixed-methods case study design has been applied, described by Creswell and Plano Clark as a methodology that ‘focuses on developing a detailed understanding of a case (or multiple cases) through gathering diverse sources of data’ (p. 116).^29^ Similarly, Ritchie and Lewis describe the primary defining features of a case study as ‘being multiplicity of perspectives which are rooted in a specific context’ (p. 52).^24^ Case studies as a method are designed to suit the case and research question, and have a level of flexibility that is not readily offered by other approaches.^32^ The use of a single case study design method in this research is predominantly due to the phenomenon being studied. The researcher has been unable to identify similar values-based Care at the End-of-Life Collaboratives in Australia, which share the same specific features, functions or focus. Having a deep understanding of the West Moreton Care at the End-of-Life Collaborative and then exploring how it can be implemented Australia-wide (in phase II of this study) will enable the first model for values-based Care at the End-of-Life Collaboratives in Australia. A mixed-methods case study methodology enables the Collaborative to be studied in context, using multiple data sources to provide a detailed description. The quantitative and qualitative data will be triangulated to verify and validate research results. For example, the results of the Partnership Self-Assessment Tool (PSAT) survey will inform the areas to be explored in the semi-structured interviews.

#### Setting

The setting for phase I of this study is the West Moreton Care at the End-of-Life Collaborative. West Moreton is a region situated approximately 50 km west of Brisbane in Queensland, Australia. The region covers a geographical area of almost 10,000 km^2^ and has a population of 373,000 people, which is expected to grow 2.6% per year for the next 25 years.^33^ Furthermore, West Moreton has several poor health and socio-economic indicators, which contribute to the growing demand for health and care services across the region. The Collaborative includes local service providers (from specialist palliative care services, to aged care services), community groups and local Government. There are approximately 30 formal members of the Collaborative, from 19 different organisations or groups. These include, but are not limited to, specialist palliative care services, acute hospital departments (e.g. intensive care), community-based health professional (GPs, nursing services), aged care services (including residential and community), universities, primary health network, community organisations, consumers and peak body organisations. There are up to a further 50 stakeholders who regularly attend Collaborative forums and events.

#### Design

**Figure 2.**
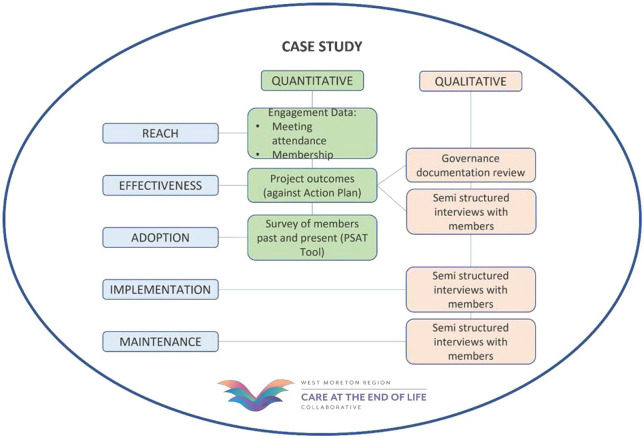
Phase I data collection.

The design of the data collection and analysis components of phase I have been developed using the RE-AIM framework, designed by Glasgow, Vogt and Boles.^34^ The framework was developed for evaluating public health interventions and includes five domains: Reach, Effectiveness, Adoption, Implementation and Maintenance/Sustainability. According to Glasgow et al., the RE-AIM framework has been applied most frequently in public health and health behaviour change research.^34^ The RE-AIM framework aligns well with the research questions for phase I of this study; however, it is noted that ‘effectiveness’ in this study refers to key success factors. Figure 2 shows the data collection methods based on the RE-AIM framework for this study.

#### Data collection

*Reach* of the Collaborative will be determined through the examination of engagement data, including membership and meeting attendance. These datasets will chronicle the development of the Collaborative and provide context regarding the size and scale of success and outcomes. These data will also show the progression of the Collaborative over time, and any changes in trends in participation, both in the number of participants and types of organisations participating. These data will be compared with the Collaborative’s individual project outcomes data to identify any correlations between member organisations and specific outcomes.

*Adoption* aspects of the RE-AIM framework will be measured through the PSAT, a valid and reliable instrument for evaluating collaborative processes.^35^ The PSAT examines six dimensions of collaboration: synergy, leadership, efficiency, administration and management, non-financial resources, financial and capital resources, and decision-making. Registered members of the Collaborative and individuals who have contributed significantly to the work of the Collaborative through participation in projects, events or planning will be invited to participate in the PSAT. These data will be used to identify the extent to which specific partnership factors are used by the Collaborative. These data will inform the design of the initial draft of a model of Care at the End-of-Life Collaborative for implementation across other jurisdictions in Australia, to be examined through a Delphi methodology in phase II of this research.

*Effectiveness* (success) of the Collaborative model will be assessed through review of project outcomes, review of governance documentation and semi-structured interviews with members and stakeholders. Analysis of the project outcomes will identify outputs and resources that are attributable to Collaborative projects, as well as data from evaluation components of these projects. These data will enable the researchers to measure the success of the Collaborative in relation to its aims, thus providing an indication of the effectiveness of the Collaborative overall.

Semi-structured interviews provide the opportunity to explore in-depth the experience of the participants, the language used and the social context of the network participants’ engagement. This methodology enables the researchers to understand the qualitative perspectives of Collaborative success in sharing the impact of networking and relationships on the Collaborative’s success in achieving its aims.

Braun and Clarke term the approach of semi-structured interviews as one that utilises a paradigmatic framework of interpretivism and constructivism to understand experience.^36^ The semi-structured interview technique enables key areas to be explored, whilst enabling the interviewer to diverge to explore emerging ideas and themes in more detail.^37^ Registered members of the Collaborative and individuals who have contributed significantly to the work of the Collaborative through participation in projects, events or planning will be invited to participate in semi-structured interviews. Participants will be recruited from the membership and affiliated specialist stakeholder lists of the Collaborative.

*Implementation* and *Maintenance* aspects of the RE-AIM framework will be explored through the semi-structured interviews. Participants will be asked about how the work of the Collaborative has influenced their organisation or individual practice. Sustainability will be addressed through questions regarding the characteristics of the Collaborative that have contributed to its sustainability to date and what factors will contribute to its future sustainability. Understanding these data is important to ensure the initial draft of the model of Care at the End-of-Life Collaborative in phase II of this research includes the critical sustainability factors identified in the semi-structured interviews.

#### Data analysis

Descriptive statistics, frequency distributions and means will be generated from the PSAT tool, using methodology recommended by PSAT tool developers. Content analysis of project and governance documents will be undertaken to determine decision-making methods and frequency, outcomes and outputs of the Collaborative. These documents include (but are not limited to) terms of reference, collaborative frameworks and meeting minutes. A narrative analysis of the Collaborative’s establishment and development will be undertaken, identifying key milestones and outputs through the Collaborative’s history, and their potential impact on outcomes. The governance of the Collaborative’s (funded) projects will also be examined to determine processes and outcomes.

A reflexive approach to thematic analysis of the semi-structured interviews will be undertaken. Audio-recorded interviews will be transcribed into a text format to enable the researchers to undertake thematic analysis. The reflexive approach was defined by Braun and Clarke and is an alternative to the ‘codebook’ approach to thematic analysis.^38^ Reflexive thematic analysis is considered a reflection of the researcher’s interpretive analysis of the data conducted at the intersection of the dataset, the theoretical assumptions of the analysis and the analytical skills of the researcher.^38^ The researcher produces themes from the data around relative core commonalities as they emerge, rather than potentially preconceived themes through predetermined coding. Braun and Clarke’s six-step model of reflexive thematic analysis as described by Byrne will be adopted in this study.^39^

### Phase II

The objective of phase II is to develop a model of Care at the End-of-Life Collaborative for implementation across other jurisdictions in Australia.

#### Setting

Representatives of each of the palliative care peak bodies in Australia (*N* = 9) will be invited to participate in phase II of this study. Participants will consider the model and implementation of Care at the End-of-Life Collaboratives in their own regions. Each organisation will be asked to nominate two representatives based on their knowledge and expertise of end-of-life care in their jurisdictions.

#### Design

A Delphi method will be used to achieve expert consensus of a Care at the End-of-Life Collaborative model, including implementation. The Delphi method is a commonly used approach to gain consensus around a given topic.^40^ It involves asking a panel of experts to anonymously provide an opinion about a specific topic, summarising the views of individuals and circulating the summary for further consideration until consensus is reached.^24^ The Delphi method was first developed in the 1950s and involves asking a panel of experts to individually provide an opinion about a specific research question/topic, summarising the views of individuals and circulating the summary for further consideration until consensus is reached.^24^ According to Barrett and Heale, the Delphi method has specific characteristics, but there can be variation in approach based on these characteristics^41^:

• There are a series of ‘rounds’ where an identified group of experts is asked their opinions on a particular issue
• The questions for each round are based on the findings of the previous round
• Experts are provided with feedback from the previous round (including their own) enabling them to reflect on their own position (in the context of others). The feedback is provided anonymously.

The Delphi method in this study is developed (and will be reported) using the Guidance on Conducting and Reporting Delphi Studies (CREDES) in palliative care, which was developed following a systematic review that showed inconsistencies in approach and methodology.^42^

#### Data collection

The Delphi surveys will be conducted online via the University of Southern Queensland’s secure Survey Tool to allow for remote participation from all jurisdictions across Australia. Participants will be asked to complete and submit their survey responses within 4 weeks from receipt. Reminders will be sent (via email) at 2 and 1 week before the submission closing date.

Based on a methodological systematic review of Delphi studies in palliative care,^42^ it is anticipated that this phase of the research will include two survey rounds. A third round will be completed if this is required to reach consensus. If, after three completed rounds, there is no consensus, the final model will be developed, omitting the elements where consensus has not been reached, with explanation of their omission, and options based on expert feedback. The first survey will detail the findings from phase I of the study and ask the experts to rate characteristics of successful Care at the End-of-Life Collaboratives, and implementation strategies, for importance. The rating will be an ascending 7-point Likert scale. The level set to determine consensus in Delphi studies has been subject to methodological criticism, with some cases being as low as 51%.^43^ Consistent with the majority of palliative care Delphi studies, consensus in this study will be a minimum of 75% scoring above 3, with a median score of 3.5 or above.^42^ The first survey will also include context-specific questions for each State and Territory of Australia.

It is anticipated that dropout rates for phase II of this study will be minimal due to the time frame anticipated for the Delphi process (6 months), the maximum of two survey rounds and the clear setting of commitment in the recruitment stage. However, should expert participants drop out, the researcher will attempt to recruit a different participant (who meets the criteria for participation) from the peak body organisation the original participant represented. Dropout rates and potential impacts of this will be reported and considered in the data analysis and interpretation of results.

#### Data analysis

Any missing data and incomplete responses from the round 1 survey will be clearly explained in the round 2 survey for the expert participants to consider in their responses. Furthermore, the quantum of missing data across the Delphi process will be considered as part of the data analysis and interpretation of results. The survey results for each round will be analysed using descriptive statistics, frequency distributions and content analysis. The results will be used to develop a model for establishing and implementing Care at the End-of-Life Collaboratives in Australia. This model will be disseminated with a second survey requesting specific feedback. The final model will be developed following the analysis of the second survey responses.

## Discussion

Studying the critical implementation and sustainability factors of the values-based interorganisational Care at the End-of-Life Collaborative in phase I of this study will inform the foundation for the consensus-based framework, which will be developed in phase II. The framework will enable the establishment of similar collaboratives throughout Australia. The need for such collaboratives is demonstrated through Australian health and palliative care activity data, which indicate that current service organisations are not meeting the needs or preferences of many Australians approaching the end of life. For example, there were 169,301 deaths in Australia in 2019, and despite the three most common underlying causes of death being coronary heart disease, dementia and cerebrovascular disease, over half (52%) of palliative care hospitalisations recorded a principal diagnosis of cancer.^44^ The Palliative Care Outcomes Collaboration (PCOC) is a collaboration of four universities that work with palliative care services in each State and Territory, to improve quality of care through benchmarking and outcome measurement. In 2021, PCOC reported that 66% of patients seen by the 177 participating palliative care services had a diagnosis of cancer^44^; further indicating the gap for people with non-cancer diagnoses in accessing specialist palliative care.

In addition to addressing service gaps, the values-based interorganisational Care at the End-of-Life Collaborative also seeks to overcome issues caused by the fragmented health system in Australia. For example, responsibilities for delivering palliative care are shared between the Australian Government and the eight State/Territory governments.^45^ The Australian Government is responsible for Medicare-funded services and aged care (primary care-based services); the State/Territory governments are responsible for public hospital-based services and some community outreach services.^46^ A study examining patients’ experiences suggests that the organisation of health services is fragmented and not in line with the principles of palliative care.^47^ The Care at the End-of-Life Collaborative model in this study uses an ecosystem approach, with the community being a critical part of the ecosystem. Integrating community capacity and resources with health and social care resources plays a role in overcoming the wicked problems caused by the fragmented health and funding systems for end-of-life care.

### Strengths and limitations

This study protocol is the first to describe a single case study based on convergent mixed-methods approach to understanding the barriers, enablers and factors that influence the success of a regional values-based interorganisational Care at the End-of-Life Collaborative. The final outputs will be a model and implementation framework for the establishment of similar Care at the End-of-Life Collaboratives in Australia, with the intention of addressing the gaps created by the fragmented care and funding systems in Australia. This study will add to the body of knowledge regarding interorganisational collaborations, specifically in the palliative care sector, where there is currently a paucity of research.

This study is focused on the collective members of the established Collaborative model and includes all members from all stakeholder groups. Whilst this approach ensures a whole of case study design is maintained, it does not represent the voice of patients, families and community alone. It is acknowledged that limitations may exist as no explicit consumer arm is presented separately in the analysis and that gaining knowledge of the Collaborative success by patients, family and community would form a separate research project for consideration.

This research is a single case study, and there has been disquiet in the literature about the validity of this design in research.^24^ However, the purpose of this research, along with the intention of a work-based project, lends itself to an in-depth examination of the complex nature of this single values-based collaborative. A mixed-methods approach has been selected to develop a detailed understanding, from multiple perspectives.

Triangulation of quantitative and qualitative data may be a validity threat to this study.^29^ Databases for qualitative and quantitative data will be kept separate and merged when complete. Results and findings from the merged data will be presented and discussed with the Collaborative members at a workshop before the commencement of phase II to further improve validity and research rigour.

The researchers acknowledge the limitations of the Delphi method identified in the literature. Expert consensus research is considered by positivist researchers to be the lowest level of evidence, with clinical trials and robust observational studies being the highest level.^42^ Despite this, the Delphi method is a validated technique for achieving consensus on a topic where scientific evidence is lacking,^48,49^ as is the case in this study. Delphi studies produce accurate and valuable results^42,50,51^ ,especially in palliative care studies where there are ethical, economic or practical barriers to using higher level research methods.^42^

## Conclusion

This protocol describes a two-phase, mixed-methods approach to examining a complex values-based Care at the End-of-Life Collaborative and developing a sustainable framework for implementation of similar models across Australia. Whilst there is an abundance of research and knowledge about interorganisational collaboratives in the health sector, there is a paucity of such research in palliative and end-of-life care. The need for more research in this specific context is derived from the many factors described in this article, the increasing demand for palliative care, the extent of community impacted by death (affecting all ages, locations, cultures and diagnoses), insufficient funding to support dying at home and the wicked problems created by a fragmented health system. This study will add to the body of knowledge regarding interorganisational collaborations in the palliative care sector and by deepening our understanding of such entities, it will be possible to design a framework for sustainable implementation across other Australian jurisdictions.
